# Preparative Purification of Liriodendrin from *Sargentodoxa cuneata* by Macroporous Resin

**DOI:** 10.1155/2015/861256

**Published:** 2015-07-07

**Authors:** Di-Hua Li, Yan Wang, Yuan-Shan Lv, Jun-Hong Liu, Lei Yang, Shu-Kun Zhang, Yu-Zhen Zhuo

**Affiliations:** ^1^Tianjin Institute of Acute Abdominal Diseases of Integrated Traditional Chinese and Western Medicine, Tianjin 300100, China; ^2^College of Pharmacy, Tianjin University of Traditional Chinese Medicine, Tianjin 300193, China

## Abstract

The preparative purification of liriodendrin from *Sargentodoxa cuneata* using macroporous resin combined with crystallization process was evaluated. The properties of adsorption/desorption of liriodendrin on eight macroporous resins were investigated systematically. X-5 resin was selected as the most suitable medium for liriodendrin purification. The adsorption of liriodendrin on X-5 resin fitted well with the pseudo-second-order kinetic model and Langmuir isotherm model. Dynamic adsorption/desorption tests were performed using a glass column packed with X-5 resin to optimize the separation process of liriodendrin. After one treatment with X-5 resin, the content of liriodendrin in the product was increased 48.73-fold, from 0.85% to 41.42%, with a recovery yield of 88.9%. 97.48% liriodendrin was obtained by further crystallization and determined by HPLC. The purified product possessed strong antioxidant activity. In conclusion, purification of liriodendrin might expend its further pharmacological researches and further applications in pharmacy.

## 1. Introduction

**Figure 1:**
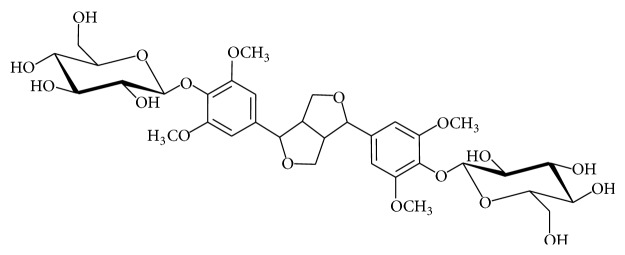
Chemical structure of liriodendrin.

*Sargentodoxa cuneata* (Lardizabalaceae) is a well-known herb of traditional Chinese medicine in China for treating abdominal pain due to acute appendicitis, sore and ulcer, dysmenorrheal, amenorrhea, traumatic swelling, and rheumatic arthritis. Modern studies have demonstrated that it possesses various pharmacological effects such as antiviral [1, 2], antibacterial [3], antioxidant [4, 5], antitumor [6], anti-inflammatory [7], resistance myocardial ischemia [8], and antithrombotic [9]. Among various active ingredients, liriodendrin (Figure 1) has been proved to be one of the bioactive compounds due to possessing of anti-inflammatory, antinociceptive [10], antiarrhythmic [11], antimyocardial ischemia [8], calcium channel antagonistic [12], inhibitory activity on HepG-2 cells [13], resisting glutamate-induced PC12 cell damage [14], increasing of heat shock factor 1 expression [15], and protecting SH-SY5Y cell effects [16].

Because of these beneficial pharmacological effects of liriodendrin, large quantities of pure compound are needed as chemical reference standard for further pharmacological researches and further applications in pharmacy. Therefore, many preparative purification methods of liriodendrin are developed, such as high-speed counter-current chromatography [17], HPLC [18], and silica gel column chromatography [10]. But these methods have several disadvantages such as having large amount of organic solvents wastage, being time consuming, having poor yield and recovery, or needing special instruments and are not suitable for large-scale industrial production. Macroporous resins are one kind of durable hydrophilic polymer with advantages of good stability, high adsorption capacity and selectivity, fast adsorption and desorption of mild conditions, easy regeneration, and saving of the cost. The principle of adsorption is based on electrostatic force, hydrogen bonding interaction, and size sieving action between macroporous resins and different molecular from the solution [19]. In recent years, macroporous resins are widely used as medium to enrich and separate the secondary metabolites of traditional Chinese medicine and nature plant, including flavonoids [20, 21], coumarins [22], terpenoids [23], lignans [24], and phenolics [25, 26].

Liriodendrin after oral administration was* in vivo* transformation to syringaresinol, which possesses strong antioxidant activity [10]. However, the antioxidant capacity of liriodendrin* in vitro* has not been investigated recently. As a part of continuing research of expanding its further applications, the antioxidant activity of liriodendrin* in vitro* was examined.

In this study, a simple method is developed for preparative purification of liriodendrin from the stem of* S. cuneata* using macroporous resin chromatography combined with crystallization process. By static and dynamic adsorption/desorption tests on resins, some process parameters, such as feed concentration and volume of sample solution, flow rate, ethanol concentration, and volume of desorption solution, are optimized for purification of liriodendrin. After further crystallization, liriodendrin is an obtained pure form and its structure is identified by NMR. Moreover, antioxidant activity of liriodendrin is also evaluated* in vitro* for the first time.

## 2. Materials and Methods

### 2.1. Materials and Reagents

Liriodendrin standard was isolated from the stem of* S. cuneata* in our previous study. The purity was determined to be over 97% by normalization of the peak areas by HPLC. Its structure was elucidated by comparison of the NMR data with the literature [27]. HPLC-grade acetonitrile and formic acid were bought from Concord Technology Co., Ltd. (Tianjin, China). 1,1-Diphenyl-2-picrylhydrazyl (DPPH) and 2,2′-azinobis (3-ethylbenzothiazoline-6-sulphonic acid) diammonium salt (ABTS) were obtained from sigma Chemical Co. (Sigma-Aldrich GmbH, Sternheim, Germany). Other chemicals and reagents were analytical grade. Deionized water was purified by a Milli-Q water purification system (Millipore, Boston, USA).

### 2.2. Adsorbents

Macroporous resins including D101, AB-8, X-5, ADS-17, and NKA were purchased from Nankai Hecheng S&T Co., Ltd. (Tianjin, China), and HPD100, HPD400, and HPD600 from Cangzhou Bon Adsorber Technology Co., Ltd. (Hebei, China), respectively. Their physical properties were summarized in Table 1. Before use, these resins were soaked in 95% v/v ethanol aqueous solutions for 24 h, then washed with 4% HCl aqueous solution, deionized water, and 4% NaOH aqueous solution, respectively, and finally washed with deionized water.

### 2.3. Preparation of Sample

The dried stem of* S. cuneata* was powdered and sieved through a number 40 mesh. The powder of sample (1.0 kg) was extracted with 8000 mL of 95% v/v ethanol aqueous solutions under reflux for 90 min and repeated twice. The extracted solutions were combined and filtered, and the filtrate was evaporated to dryness by a rotary evaporator under reduced pressure at 60°C. The residue was suspended in deionized water and suspended solution was centrifuged at 3000 rpm for 30 min. The supernatant was used as stock solution for the subsequent test.

### 2.4. HPLC Analysis of Liriodendrin

The HPLC system consisted of a SSI PC2000 chromatograph (SSI, Scientific Systems, Inc., USA) and a 2000ES evaporative light scattering detector (ELSD) (Alltech, Alltech Associates Inc., USA). An ODS-2 Hypersil C-18 column (250 mm × 4.6 mm id, 5 *μ*m; Thermo Scientific, Thermo Fisher Scientific Inc., USA) was operated at 35°C. Solvents that constituted the mobile phase were acetonitrile (A) and 0.2% formic acid aqueous solution (B). The separation was performed using a stepwise gradient elution of 0–14 min, 95–87% B; 14–25 min, 87–87% B; and 25–45 min, 87–81% B; then keeping 95% B to balance for 10 min. The flow rate was 0.9 mL/min. ELSD was used as the detection method with nebulizing gas flow rate of 2.5 L/min and drift tube temperature of 105°C. The impactor position of ELSD was set off. The stock standard solution of liriodendrin was prepared and diluted six concentrations of 37–370 *μ*g/mL. It showed a good linearity over range of 0.74–7.4 *μ*g for liriodendrin. The regression equation for liriodendrin was *Y* = 1.63681*X* + 5.40736 (*R* ^2^ = 0.9992, *n* = 6), where *Y* is the logarithm peak area and *X* is the logarithm mass of liriodendrin.

### 2.5. Static Adsorption and Desorption Tests

#### 2.5.1. Adsorption Resins Screening

All macroporous resins were screened through static adsorption test. Pretreated resin (equivalent to 1 g dry resin) was put into a 50 mL Erlenmeyer flask with stopper and 10 mL aqueous solution of* S. cuneata* extracts in Section 2.3 was added. The flasks were shaken (180 rpm) for 8 h at 25°C. After adsorption equilibrium was reached, resins were washed by deionized water. Then the resins were desorbed with 10 mL 95% v/v ethanol aqueous solutions and the flasks were shaken (180 rpm) for 8 h at 25°C. The solutions after adsorption and desorption were determined by HPLC. The experiments were repeated three times for each resin. The following equations were used to calculated adsorption capacity, adsorption ratio, and desorption ratio.

Adsorption capacity is

(1) $$\begin{array}{c} Q_{e}=\frac{V_{i}\left(C_{0}-C_{e}\right)}{W}. \end{array}$$

Adsorption ratio is

(2) $$\begin{array}{c} E=\frac{C_{0}-C_{e}}{C_{0}}\times 100\%, \end{array}$$

where *Q*_*e*_ is the adsorption capacity at the adsorption equilibrium (mg/g dry resin); *E* is the adsorption ratio (%); *V*_*i*_ is the initial volume of feed solution (mL); *C*_0_ and *C*_*e*_ are the initial and equilibrium concentration of liriodendrin in solutions, respectively (mg/mL); and *W* is the weight of the dry resin (g).

Desorption ratio is

(3) $$\begin{array}{c} D=\frac{C_{d}V_{d}}{V_{i}\left(C_{0}-C_{e}\right)}\times 100\%, \end{array}$$

where *D* is the desorption ratio (%); *C*_*d*_ is the concentration of liriodendrin in the desorption solution (mg/mL); *V*_*d*_ is the volume of the desorption solution (mL); *V*_*i*_, *C*_0_, and *C*_*e*_ are the same as defined above.

#### 2.5.2. Adsorption Kinetics

The adsorption kinetics of D101 and X-5 resins were studied by mixing 10 mL aqueous solution of* S. cuneata* extracts (the initial concentration of liriodendrin 2.131 mg/mL) with pretreated resin (1 g dry resin) in each 50 mL Erlenmeyer flask with stopper and shaken (180 rpm) at 25°C. Liriodendrin concentration in the solution phase was monitored by HPLC at different time intervals until equilibrium. To examine the underlying mechanism of the adsorption kinetic process, equations of pseudo-first-order model, pseudo-second-order model, and intraparticle diffusion model were used to describe adsorption process [23].

Equation of pseudo-first-order kinetic model is

(4) $$\begin{array}{c} \ln\left(\;Q_{e}-Q_{t}\;\right)=\ln Q_{e}-k_{1}t. \end{array}$$

Equation of pseudo-second-order kinetic model is

(5) $$\begin{array}{c} \frac{t}{Q_{t}}=\frac{t}{Q_{e}}+\frac{1}{{Q_{e}}^{2}k_{2}}. \end{array}$$

Equation of intraparticle diffusion kinetic model is

(6) $$\begin{array}{c} Q_{t}=k_{i}t^{0.5}+C, \end{array}$$

where *Q*_*e*_ and *Q*_*t*_ are the adsorption capacity at equilibrium and at any time *t* (mg/g dry resin), respectively; *k*_1_ (min^−1^), *k*_2_ (g/mg·min), and *k*_*i*_ (mg/g·min^0.5^) are the rate constant for the adsorption process; *C* is the constant, indicating the boundary layer thickness (mg/g).

#### 2.5.3. Adsorption Isotherms

The equilibrium adsorption isotherms of liriodendrin on D101 and X-5 resins were studied by the following experiment. D101 or X-5 pretreated resin (1 g dry resin) and 10 mL aqueous solution of* S. cuneata* extracts (the initial concentration of liriodendrin at 0.213, 0.426, 0.852, 1.279, 1.705, and 2.131 mg/mL) were added into a 50 mL Erlenmeyer flask with stopper, respectively, and shaken (180 rpm) for 8 h at 25°C. The equilibrium concentration of liriodendrin was analyzed. For further explanation of how molecules or ions of adsorbate interact with adsorbent surface site, the equilibrium adsorption isotherms of D101 and X-5 resins were described by Freundlich and Langmuir equations, respectively. Freundlich equation described the adsorption behavior of the monomolecular layer as well as multimolecular layer and Langmuir equation only described the monolayer adsorption [28, 29].

Freundlich equation is

(7) $$\begin{array}{c} Q_{e}=K_{F}C_{e}^{1/n}. \end{array}$$

Langmuir equation is

(8) $$\begin{array}{c} Q_{e}=\frac{Q_{0}K_{L}C_{e}}{1+K_{L}C_{e}}, \end{array}$$

where *K*_*F*_ is the Freundlich constant, an indicator of adsorption capacity; 1/*n* is an empirical constant related to magnitude of the adsorption driving force; *Q*_0_ is the theoretical maximum adsorption capacity (mg/g dry resin); *K*_*L*_ is the Langmuir adsorption equilibrium constant; *Q*_*e*_ and *C*_*e*_ are the same parameters as in (1).

### 2.6. Dynamic Adsorption and Desorption Tests

Dynamic adsorption/desorption tests were performed with a glass column (20 mm × 300 mm) wet-packed of X-5 resin (6 g dry resin). The bed volume (BV) was 30 mL. For the dynamic breakthrough experiment, aqueous solution of* S. cuneata* extracts was loaded into the glass column at a prescribed flow rate (1, 2, and 4 BV/h), respectively. The liriodendrin concentrations in the effluents that were collected at 10 mL intervals were determined by HPLC.

For dynamic desorption experiment, aqueous solution of* S. cuneata* extracts was loaded into the resin column mentioned above. After adsorption equilibrium, the resin column was washed first with 2 BV deionized water, and then desorption was conducted with different concentrations of ethanol aqueous solutions (10, 20, 30, 40, 50, 60, 70, 80, and 95% v/v) at a flow rate of 1 BV/h successively. The volume of each ethanol aqueous solution was 3 BV and each ethanol desorption solution was analyzed by HPLC. For the quantity of ethanol elution, the test was performed as follows: when the adsorption equilibrium was reached, the resin column was washed first with 2 BV deionized water and then was eluted with 6 BV 40% v/v ethanol aqueous solutions at a flow rate of 1 BV/h. The elution was collected at 1 BV intervals and determined by HPLC. All the 40% v/v ethanol elution was collected and concentrated to dryness under reduced pressure at 60°C to give refined product.

### 2.7. Crystallization of Liriodendrin

The refined product was extracted with methyl alcohol-chloroform (1 : 4, v/v) under reflux for 40 min and repeated twice. The extracted solutions were combined and filtered, and the filtrate was concentrated to dryness under reduced pressure at 60°C. The residue was dissolved in methyl alcohol (1 : 10, w/v). After being kept at 20–30°C for several days, the solution was centrifuged to obtain crystals of liriodendrin. Then crystals were washed by methyl alcohol.

### 2.8. Antioxidant Activity

#### 2.8.1. DPPH Radical Scavenging Assay

The DPPH radical scavenging activity of samples was evaluated by the method reported [30]. Serial dilutions of the liriodendrin in methanol (1 mL) were added to 4 mL methanol solution of DPPH (0.1 mM/L). After 30 min, the absorbance was measured at 517 nm against a control at room temperature. Vitamin C was used as a positive. The DPPH radical scavenging activity was calculated using the following equation:

(9) DPPH∙  scavenging  effect%=Acontrol−AsampleAcontrol×100.

#### 2.8.2. ABTS^•+^ Scavenging Assay

ABTS^•+^ scavenging assay was performed by the literature method [30]. The ABTS^•+^ solution was generated by the interaction of ABTS (7 mM/L) and potassium persulfate (2.45 mM/L), stored in the dark at room temperature for 12 h. The ABTS^•+^ solution was diluted until absorbance was 0.700 ± 0.025 at 734 nm with ethanol. Then, serial dilutions of the liriodendrin in ethanol (1 mL) were added to the ABTS^•+^ solution (4 mL). After 30 min, the absorbance was measured at 734 nm against a control at room temperature. Vitamin C was used as a positive. The scavenging capability of ABTS^•+^ was calculated using the following equation:

(10) ABTS∙+  scavenging  effect%=Acontrol−AsampleAcontrol×100.

## 3. Results and Discussion

### 3.1. Resins Screening

The selection of proper macroporous resins was performed in accordance with the structures and polarities of adsorbates and resins [23]. In the paper, eight macroporous resins of different properties were studied at 25°C. As shown in Table 2, macroporous resins of nonpolar and weakly polar exhibited better adsorption capacity and desorption ratios than others. This may be caused for special structure of liriodendrin. Although liriodendrin molecule contained polar glucuronyl group, the symmetrical structure of liriodendrin make it possess feature of nonpolar or weakly polar. Therefore, nonpolar and weakly polar resins were applicable to adsorption of liriodendrin. Adsorption capacity and adsorption ratios of D101 and X-5 resins were higher than other resins, and desorption ratios of AB-8, D101, and X-5 resins were similar and higher than other resins. Therefore, D101 and X-5 resins were selected to further evaluate for their properties in the following experiment.

### 3.2. Adsorption Kinetics

**Figure 2:**
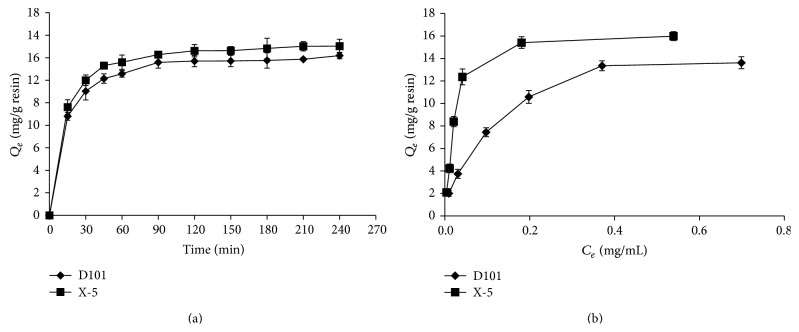
Adsorption kinetics curves (a) and adsorption isotherms curves (b) for liriodendrin on D101 and X-5 resins at 25°C.

Adsorption kinetics on D101 and X-5 resins were evaluated at 25°C. Adsorption kinetic curves were shown in Figure 2(a). For the two resins, the adsorption capacities toward liriodendrin increased rapidly in the 30 min, and an asymptotic curve was reached at about 120 min. The fast initial adsorption was likely due to rapid attachment of liriodendrin to the surface of resin and an asymptotic period due to diffusion of liriodendrin into the micropores of resin with high intraparticle mass transfer resistance, which indicated that the behaviors of two resins belonged to the fast adsorption resin type [31]. The parameters of three kinetic models were shown in Table 3(a). According to the calculated correlation coefficient (*R* ^2^ ≥ 0.99), the pseudo-second-order kinetic model performed better to describe the adsorption process on X-5 and D101 resins, which suggested that the adsorption rate was controlled by chemical adsorption mechanism through sharing or exchange of electrons between adsorbate and adsorbent [32]. In terms of the rate constant *k*_2_, the adsorption rate of X-5 and D101 resin was the same.

### 3.3. Adsorption Isotherms

Adsorption isotherms curves were obtained for liriodendrin on D101 and X-5 resins at 25°C. As shown in Figure 2(b), adsorption capacities increased with increasing initial concentration, and a turning point was observed when the initial liriodendrin concentration was 1.705 mg/mL. Therefore, this concentration of liriodendrin in sample solution was selected in the following test. Table 3(b) listed the two isotherms equations and relative parameters. The calculated correlation coefficients were shown that the Langmuir model fitted the test data more suitable than the Freundlich model in the studied concentration range, which suggested the monolayer coverage of liriodendrin on the resin [22, 28]. The theoretical maximum adsorption capacities *Q*_0_ on D101 and X-5 resins, determined from the Langmuir equation, were 15.4321 mg/g and 16.7504 mg/g. Therefore, X-5 resin was selected for dynamic adsorption and desorption tests.

### 3.4. Dynamic Adsorption-Desorption Tests

#### 3.4.1. Dynamic Breakthrough Curves on X-5 Resin

**Figure 3:**
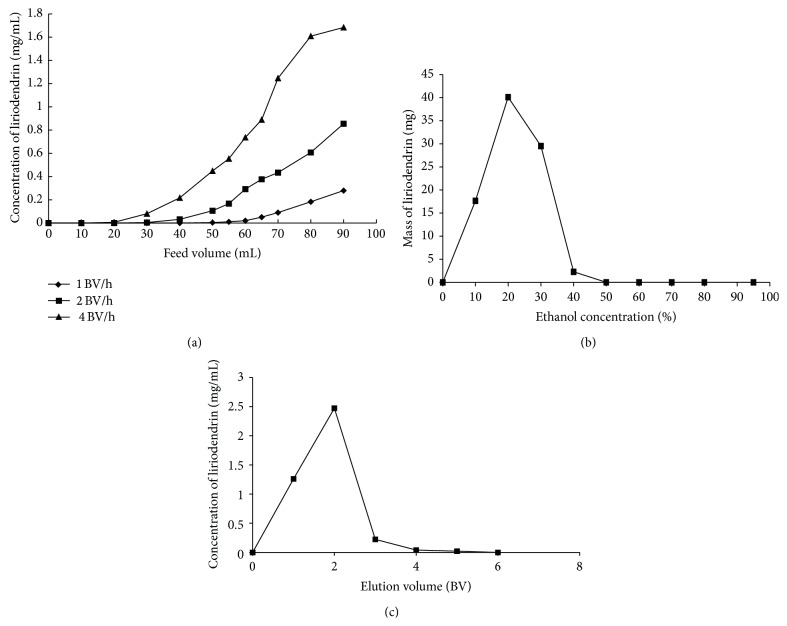
Dynamic adsorption and desorption test curves on X-5 resin. (a) Dynamic leakage curve, (b) gradient elution curve, and (c) isocratic desorption curve of liriodendrin on X-5 resin column at 25°C.

Dynamic breakthrough tests were studies based on the feed concentration, the volume of effluent, flow rate, and temperature. The initial feed concentration of liriodendrin was 1.705 mg/mL (from Section 3.3) and the temperature at 25°C, and then the effect of flow rate was investigated. As shown in Figure 3(a), the different flow rates showed noticeable different breakthrough volume. When the flow rate was 1 BV/h, the best adsorption performance could be obtained. The breakthrough volume of liriodendrin on X-5 resin was 55 mL (about 2 BV) at a flow rate of 1 BV/h, when the liriodendrin concentration in effluents reached 1% of the initial concentration [18]. The adsorption capacity of liriodendrin on X-5 resin was 15.630 mg/g dry resin.

#### 3.4.2. Dynamic Desorption on X-5 Resin

In the process of dynamic desorption, the concentration and the volume of desorption solution were investigated. Dynamic desorption was performed with gradient and isocratic elution modes at the flow rate of 1 BV/h, respectively. As shown in Figure 3(b), the desorption ability increased by the increase of ethanol concentration. When the concentration of ethanol reached 40% v/v, the liriodendrin absorbed by X-5 resin could be fully eluted. Therefore, 40% v/v ethanol aqueous solution was selected as the eluent in isocratic elution. The isocratic elution result showed that the concentration of liriodendrin reached maximum values when eluting with 2 BV of 40% v/v ethanol aqueous solutions. When the volume of elution reached 5 BV, liriodendrin could be completely desorbed from X-5 resin (Figure 3(c)). Therefore, the volume of desorption solution was selected to be 5 BV. Under the above optimized condition, all the 40% v/v ethanol elution was collected and concentrated to dryness under reduced pressure at 60°C to give refined product.

### 3.5. Purity Analysis and Identification of Liriodendrin

**Figure 4:**
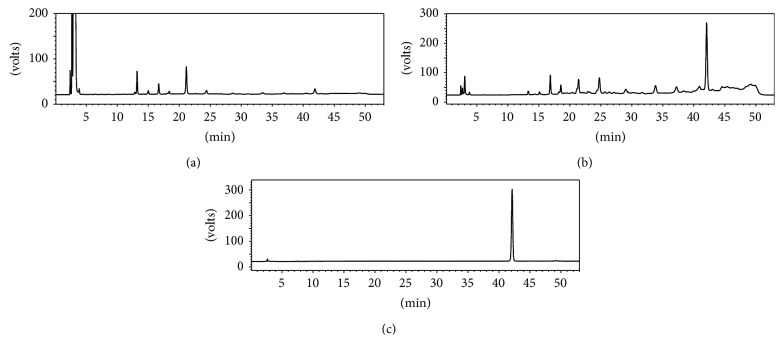
HPLC chromatograms of samples before treatment (a) and after treatment (b) on X-5 resin; liriodendrin purified by crystallization (c).

After purification on X-5 resin, the purity of liriodendrin increased from 0.85% of* S. cuneata* extracts to 41.42%, and the liriodendrin of recovery rate was 88.9%. Such crystallization resulted in a product with 97.48% purity and the recovery rate was 63.7% (Table 4). As shown in Figures 4(a) and 4(b), the HPLC chromatograms of* S. cuneata* extracts before and after treatment on X-5 resin were compared.  Figure 4(c) showed that the purity of liriodendrin crystals was determined to be 97.48% by HPLC. For further chemical structure identification of liriodendrin, NMR analysis was performed and the data was shown as follows: ^1^H-NMR (DMSO-d_6_, 400 MHz) *δ*: 6.65 (4H, s, H-2, 2′, 6, 6′), 4.93 (2H, d, *J* = 6.8 Hz, H-1′′), 4.66 (2H, d, *J* = 3.6 Hz, H-7, 7′), 4.31 (2H, m, Hb-9, 9′), 4.19 (2H, m, Ha-9, 9′), 3.75 (12H, s, 4 × OCH_3_); ^13^C-NMR (DMSO-d_6_, 100 MHz) *δ*c: 152.6 (C-3, 3′, 5, 5′), 137.1 (C-4, 4′), 133.8 (C-1, 1′), 104.3 (C-2, 2′, 6, 6′), 102.7 (C-1′′), 85.0 (C-7, 7′), 77.2 (C-5′′), 76.5 (C-3′′), 74.1 (C-2′′), 71.3 (C-9, 9′), 70.0 (C-4′′), 60.9 (C-6′′), 56.4 (-OCH_3_), 53.6 (C-8, 8′). The data were identical with those reported in the literature [27].

### 3.6. Antioxidant Activity

The antioxidant effect of plant samples can be evaluated by several* in vitro* tests. Since the assay results of the antioxidant effect depend on the method used, a combined assay of several methods is required. In this study, the antioxidant activity of liriodendrin isolated from* S. cuneata was* analysed using DPPH and ABTS.

**Figure 5:**
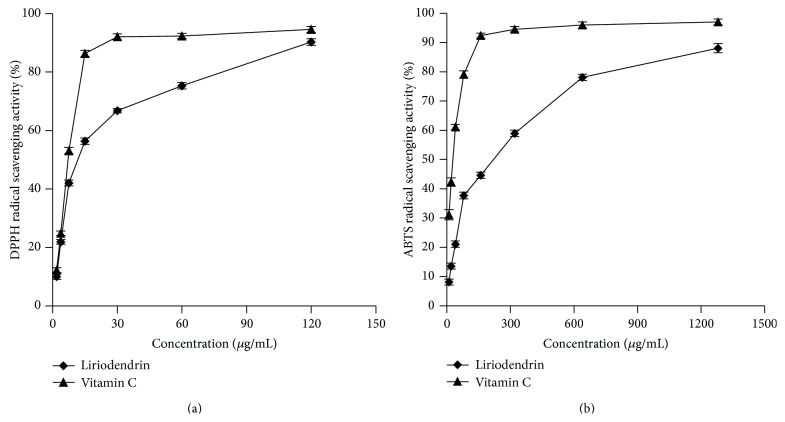
DPPH radical scavenging activity (a) and ABTS^•+^ scavenging assay (b).

DPPH radical scavenging activity test aims to measure the capacity of sample to scavenge the stable radical DPPH formed in solution by donation of hydrogen atom or an electron [33]. As shown in (Figure 5(a)), the IC_50_ value of liriodendrin is 13.61 *μ*g/mL, which is less important than that of vitamin C (IC_50_ = 6.98 *μ*g/mL).

ABTS^•+^ scavenging assay is a widely accepted model to determine the total antioxidant activity. As shown in (Figure 5(b)), the IC_50_ values of liriodendrin and vitamin C are 168.42 and 23.29 *μ*g/mL, respectively.

The ABTS^•+^ scavenging assay revealed that liriodendrin possessed weak ABTS^•+^ scavenging capacities. However, it has been suggested that liriodendrin plays an important role in DPPH inhibitory activities and oxygen radical absorbance capacities. On the other hand, liriodendrin after oral administration was* in vivo* transformation to syringaresinol, which possesses strong antioxidant activity [10]. The antioxidant activity of liriodendrin* in vitro* and* vivo* needed to be further researched.

## 4. Conclusions

In the study, highly concentrated liriodendrin was successfully prepared. After* S. cuneata* extracts were purified on X-5 resin, purity of liriodendrin was 41.42%. Further crystallization resulted in a product with 97.48% purity and the recovery rate was 63.7%. The results indicated that the established method was simple and effective, thus showing potential for industrial scale isolation of liriodendrin from* Sargentodoxa cuneata* in the future. Also, such production of highly concentrated liriodendrin as an antioxidant agent might expand its further applications both in industrial production and in pharmacy.

## Figures and Tables

**Table 1:** Physical properties of macroporous resins in test.

Resin	Average pore diameter (nm)	Surface area (m^2^/g)	Polarity
AB-8	13-14	450–530	Weakly polar
D101	10–12	600–700	Nonpolar
NKA	20–22	570–590	Nonpolar
X-5	29-30	500–600	Nonpolar
ADS-17	25–30	90–150	Middle-polar
HPD100	8.5–9	650–700	Nonpolar
HPD400	7.5–8	500–550	Middle-polar
HPD600	8	550–600	Strong-polar

**Table 2:** Adsorption capacity and adsorption and desorption ratios of liriodendrin on different resins at 25°C.

Resin	Adsorption capacity^a^ (mg/g)	Adsorption ratios^a^ (%)	Desorption ratio^a^ (%)
AB-8	14.206 ± 0.167	83.33 ± 0.98	92.45 ± 0.49
D101	14.535 ± 0.282	85.26 ± 1.66	91.65 ± 0.39
NKA	12.299 ± 0.176	72.14 ± 1.03	83.27 ± 0.83
X-5	15.870 ± 0.135	93.09 ± 0.79	92.88 ± 0.37
ADS-17	10.024 ± 0.267	58.96 ± 1.21	75.41 ± 0.88
HPD100	14.038 ± 0.102	82.34 ± 0.60	87.98 ± 0.41
HPD400	11.157 ± 0.187	65.45 ± 1.66	82.30 ± 1.91
HPD600	9.219 ± 0.187	54.08 ± 1.10	89.53 ± 0.81

^a^Values are means ± SD (*n* = 3).

**Table:** (a) Kinetic parameters of liriodendrin on D101 and X-5 resins at 25°C

Resin	D101	X-5
Pseudo-first-order		
Equation	ln⁡(13.6468 − *Q*_*t*_) = 2.6135 − 0.0596*t*	ln⁡(14.5831 − *Q*_*t*_) = 2.6799 − 0.0634*t*
*R* ^2^	0.9883	0.9900
*k*_1_ (min^−1^)	0.0596	0.0634
*Q*_*e*_ (mg/g)	13.6468	14.5831

Pseudo-second-order		
Equation	*t*/*Q*_*t*_ = 0.0694*t* + 0.4759	*t*/*Q*_*t*_ = 0.0650*t* + 0.4177
*R* ^2^	0.9988	0.9991
*k*_2_ (g/mg·min)	0.0101	0.0101
*Q*_*e*_ (mg/g)	14.4092	15.3846

Intraparticle diffusion		
Equation	*Q*_*t*_ = 0.7261*t* ^0.5^ + 4.9887	*Q*_*t*_ = 0.7662*t* ^0.5^ + 5.4919
*R* ^2^	0.7080	0.6936
*k*_*i*_ (mg/g·min^0.5^)	0.7261	0.7662
*C* (mg/g)	4.9887	5.4919

**Table:** (b) Isotherm parameters of liriodendrin on D101 and X-5 resins at 25°C

Resin	D101	X-5
Freundlich		
Linear equation	ln⁡*Q*_*e*_ = 0.4731ln⁡*C*_*e*_ + 2.9920	ln⁡*Q*_*e*_ = 0.4069ln⁡*C*_*e*_ + 3.3704
*K*_*F*_	19.9255	29.0902
1/*n*	0.4731	0.4069
*R* ^2^	0.9701	0.8236

Langmuir		
Linear equation	*C*_*e*_/*Q*_*e*_ = 0.0648*C*_*e*_ + 0.0054	*C*_*e*_/*Q*_*e*_ = 0.0597*C*_*e*_ + 0.0014
*Q*_0_ (mg/g)	15.4321	16.7504
*K*_*L*_	12.0000	42.6429
*R* ^2^	0.9948	0.9988

**Table 4:** The purities and recoveries of liriodendrin in the two-step purification.

Step	Purity (%)	Recovery (%)	Yield (mg)
Crude extract	0.85	—	—
X-5 resin	41.42	88.9	201.3^a^
Crystallization	97.48	63.7	270.7^b^

^a^The amount of refined sample was obtained from 55 g raw material in the X-5 resin treatment.

^b^The amount of liriodendrin was obtained from 1000 mg refined sample by crystallization.
